# Association of early versus late tracheostomy with prognosis in hypoxic‐ischaemic encephalopathy patients: A propensity‐matched cohort study

**DOI:** 10.1111/nicc.13268

**Published:** 2025-02-26

**Authors:** Yeling Li, Dingyuan Wan, Hongmei Liu, Keying Guo, Yilin Liu, Lihong Zhao, Ming Li, Jijie Li, Yiwen Liu, Wei Dong

**Affiliations:** ^1^ Department of Critical Care Medicine, West China Hospital, Sichuan University/West China School of Nursing Sichuan University Chengdu China; ^2^ Sichuan University Chengdu China; ^3^ Department of Respiratory, West China Hospital Sichuan University Chengdu China; ^4^ Department of Radiology, West China Hospital, Sichuan University/West China School of Nursing Sichuan University Chengdu China; ^5^ Department of Neurology, West China Hospital, Sichuan University/West China School of Nursing Sichuan University Chengdu China; ^6^ West China School of Public Health, West China Second Hospital Sichuan University Chengdu China; ^7^ Department of Critical Care Medicine, West China Hospital Sichuan University Chengdu China

**Keywords:** hypoxic‐ischaemic encephalopathy, mechanical ventilation, prognosis, propensity score matching, tracheostomy

## Abstract

**Background:**

The optimal timing for exchanging an endotracheal tube for a tracheostomy cannula in patients with hypoxic‐ischaemic encephalopathy is controversial.

**Aim:**

This study aimed to evaluate the effects of early versus late tracheostomy on the prognosis of patients with hypoxic‐ischaemic encephalopathy.

**Study Design:**

The study was an observational retrospective study that followed the Strengthening the Reporting of Observational Studies in Epidemiology guidelines. We included adults with hypoxic‐ischaemic encephalopathy who underwent tracheostomy between January 2012 and September 2020. The patients were classified into early or late tracheostomy groups. To eliminate differences in baseline characteristics, propensity score matching was conducted, and the outcomes between the two groups were compared.

**Results:**

A total of 132 patients were included, and through propensity score matching, 54 pairs of patients were matched. The early tracheostomy group showed a significant reduction in the duration of mechanical ventilation (median, 12 days; interquartile range 7–20 vs. median, 28 days; interquartile range, 15.75–58.25, *p* < .001), intensive care unit length of stay (median, 14.5 days; interquartile range, 6.75–26 vs. median, 35 days; interquartile range, 20–59, *p* < .001) and hospital length of stay (median, 19.5 days; interquartile range, 10.87–36.5 vs. median, 39.5 days; interquartile range, 22–66, *p* < .001). Over a 1‐year follow‐up period, there were no significant differences between the two groups regarding inhospital mortality (57.4% vs. 46.3%, *p* = .248), 30‐day mortality (59.3% vs. 46.3%, *p* = .177) and 1‐year mortality (61.1% vs. 48.1%, *p* = .176).

**Conclusions:**

In patients with hypoxic‐ischaemic encephalopathy undergoing mechanical ventilation, early tracheostomy is associated with a reduction in the duration of mechanical ventilation and decreased intensive care unit and hospital length of stay.

**Relevance to Clinical Practice:**

For patients with hypoxic‐ischaemic encephalopathy who are at a high risk of requiring prolonged mechanical ventilation, the benefits of early tracheostomy suggest considering it a viable treatment option.

**What is known about the topic**

- For individuals with hypoxic‐ischaemic encephalopathy, tracheostomies serve as a critical option for long‐term support in mechanical ventilation scenarios.
- The optimal timing for performing a tracheostomy in the context of hypoxic‐ischaemic encephalopathy remains a contentious issue within the medical community, with significant implications for patient care and recovery.

**What this paper adds**

- Early tracheostomy in patients with hypoxic‐ischaemic encephalopathy can significantly reduce the duration of mechanical ventilation and the overall hospital stay.
- The timing of tracheostomy in patients with hypoxic‐ischaemic encephalopathy does not appear to be significantly correlated with a reduction in the incidence of ventilator‐associated pneumonia or improvements in mortality rates.

## 1 INTRODUCTION AND BACKGROUND

Hypoxic‐ischaemic encephalopathy (HIE) is a brain condition brought on by an inadequate blood and oxygen supply because of a variety of causes. It is present in approximately 20% of patients with brain injury and is typically associated with poor neurological functional outcomes.^1^ There are multiple causes of HIE in adults, the most common being inadequate cerebral blood perfusion because of cardiac and respiratory arrest, which leads to secondary hypoxia and brain damage.^2^ Severe intracranial hypertension, carbon monoxide poisoning, diffuse cerebral vasospasms, shock and status epilepticus are further causes of HIE.^3^ Only 22% of inpatients and 6% of outpatients with cardiac arrest who undergo treatment for HIE survive to be discharged.^4^, ^5^ Mechanical ventilation (MV), an advanced life support measure, is applied to patients with HIE to prolong their survival.^6^

The most common recommendation for a tracheostomy is to provide a long‐term airway for patients with HIE who require prolonged MV.^7^ Tracheostomy has potential benefits, including promoting oral and airway hygiene, enhancing patient comfort, reducing the use of sedative drugs, decreasing the duration of MV and improving patient communication and swallowing abilities.^8^, ^9^ However, routine tracheostomy may inadvertently increase the risk of exposure to certain complications for some patients, including local bleeding, pneumothorax, infection and recurrent laryngeal nerve injury.^10^ It may also increase the occurrence of ventilator‐associated pneumonia (VAP).^11^, ^12^

Despite the perceived benefits of tracheostomy in MV, the medical community has yet to reach a consensus on the optimal timing for this procedure. Traditional intervention measures are performed when the duration of endotracheal intubation in patients exceeds 10–14 days. A tracheostomy performed within 10 days is referred to as early tracheostomy (ET).^13^ According to several large‐scale multicentre randomized controlled trials (RCTs),^12^, ^14^, ^15^ ET is not associated with better outcomes. However, an increasing number of sizeable systematic review studies^7^, ^16^, ^17^, ^18^, ^19^, ^20^ suggest potential benefits of ET, such as lower mortality rate, less mean time spent on MV and shorter hospitalization duration. An observational study^21^ in the United States on patients with trauma found that ET significantly reduced the incidence of pulmonary complications and the utilization of critical care resources. A study^22^ conducted in China has demonstrated that ET can significantly reduce the duration of hospitalization and lower the treatment costs associated with intensive care unit (ICU) stays for patients with severe stroke. However, RCTs^9^, ^15^ have not demonstrated significant short‐term or long‐term mortality benefits associated with ET. Moreover, data on the optimal timing of tracheostomy in patients with HIE are limited.

## 2 AIMS

For patients anticipated to require extended MV, the ongoing controversy over the optimal timing for tracheostomy can result in considerable variability in clinical practice. This variability can significantly influence patient quality of life, as well as incur substantial costs to the health care system and society at large.^23^ In addition, including heterogeneous patient‐case combinations in studies on the optimal timing of tracheostomy may mask the potential benefits of ET. Thus, the impact of ET and late tracheostomy (LT) on the prognosis of patients with HIE receiving MV remains unclear. Therefore, this study aimed to evaluate the prognostic effects of ET and LT in patients with HIE and further explore the optimal timing for tracheostomy. To elucidate the association of ET and LT with prognosis in HIE, propensity score matching (PSM) was conducted.

## 3 DESIGN AND METHODS

### 3.1 Study design

This retrospective observational cohort study adhered to the Strengthening the Reporting of Observational Studies in Epidemiology (STROBE) guidelines.^24^

### 3.2 Setting and sample

This study examined patients with HIE who underwent tracheostomies between January 2012 and September 2020. The inclusion criteria were as follows: (1) a diagnosis code of 348.1 (Anoxic brain injury)^25^ from the International Classification of Diseases, Ninth Revision, Clinical Modification (ICD‐9‐CM), which indicates a definite history of cerebral hypoxia and diffuse indications of cerebral damage or suffering cardiac arrest; (2) age ≥18 years; and (3) receipt of tracheostomy following endotracheal intubation while hospitalized. The exclusion criteria were as follows: (1) anticipated requirement for a surgical tracheostomy that would require ongoing care; (2) pregnancy; (3) involvement in any other interventional trial; and (4) death within the first 72 h.

The patients were divided into two groups: an ET group (tracheostomy performed within 10 days after endotracheal intubation) and an LT group (tracheostomy performed 10 days after endotracheal intubation) based on a Cochrane systematic review.^7^

### 3.3 Sample size

In this study, the hospital length of stay (LOS) was the primary outcome measure. Based on the results of analogous published research,^26^ we utilized PASS 2021 software to calculate the required sample size,^27^ determining that at least 102 participants should be enrolled. This strategy ensures the scientific validity of our research design.

### 3.4 Ethical and research approvals

This study was approved by the ethics committee and conducted in accordance with the Declaration of Helsinki. Given the non‐interventional and observational nature of the research, the ethics committee reviewed and waived the requirement for obtaining patient consent.

### 3.5 Procedure, indications and timing of tracheostomy

The tracheostomies performed on the participants in this study adhered to the established routine procedures of the hospital. Under local anaesthesia, both percutaneous and traditional tracheostomies were carried out. To separate the skin and subcutaneous tissues from the front of the trachea, a midline incision was made along the anterior neck. A properly sized endotracheal tube with a core was inserted after the trachea was cut open. The inner tube was inserted right after the outer tube was implanted, and the core was taken out. After aspirating the secretions, the presence of bleeding was determined.

Finally, the neck was secured with the strap. Following a thorough evaluation of patients with HIE regarding the extent of injury, expected duration of MV, arterial blood gas analyser results and smoking history, a tracheostomy was carried out. Furthermore, at least one of the following conditions served as the primary basis for tracheostomy indications: (1) continuous need for suctioning bronchotracheal secretions; (2) central nervous system‐related respiratory insufficiency; and (3) aspiration or risk of aspiration owing to dysphagia.^28^

### 3.6 Data collection

Clinical information was recorded from medical charts. The following data were collected: sex, age, weight, primary disease, Acute Physiology and Chronic Health Evaluation II (APACHE‐II) score^29^ at hospital admission, Glasgow Coma Scale (GCS) score at hospital admission, Pittsburgh Cardiac Arrest Category (PCAC)^30^ at hospital admission, LOS before ICU admission, the time between onset and resumption of spontaneous circulation, tracheostomy before ICU admission, types of ICU, causes of heart attack, the site of the heart attack, intubation during cardiopulmonary resuscitation, thrombolytic therapy before hospital admission, hypothermia treatment before ICU admission, hypothermia treatment before hospital admission, coronary angiography before ICU admission, tracheostomy approach and the surgical site. The laboratory test data within 24 h of hospital admission included haemoglobin content, blood platelet count, creatinine content, urea nitrogen content and albumin content. The study employed a 1‐year follow‐up period to evaluate the long‐term outcomes of the patients enrolled.

### 3.7 Outcomes

The primary clinical outcomes were as follows: (1) hospital LOS, measured as the time from hospital admission to discharge or death (days); (2) duration of MV, measured as the time from endotracheal intubation to extubation (days); and (3) ICU LOS, defined as the time from a patient's first ICU transfer or admission to the occurrence of death in the ICU, hospital discharge or transfer out of the ICU (days).

The secondary outcomes included: (1) incidence of VAP; (2) successful weaning from the ventilator following a tracheostomy; (3) inhospital mortality; (4) discharge status; (5) 30‐day all‐cause mortality; and (6) 1‐year all‐cause mortality. Data were extracted and analysed by two operators who were blinded to the aim of the study.

The diagnostic criteria for VAP in this study are based on the following standards^31^: the presence of a new or progressively enlarging pulmonary infiltrate detectable via chest x‐ray or CT scan within 48 h of initiating or discontinuing MV. Furthermore, at least one of the following clinical indicators must accompany this finding: signs of pulmonary consolidation and/or moist rales; an elevated peripheral leukocyte count (WBC > 10.0 × 10^9^/L); a body temperature exceeding 38°C; purulent secretions in the airway; or the isolation of a novel pathogen from respiratory secretions.

### 3.8 Statistical analysis

Descriptive statistics, *t*‐tests, Mann–Whitney *U* tests, chi‐square (χ^2^) tests and the Kaplan–Meier method were performed using SPSS software, version 25.0 (IBM, Chicago, IL, USA). Continuous variables with a normal distribution were reported as mean with standard deviation (SD) or median and interquartile range (IQR), while categorical variables were expressed as counts (n) and percentages (%). For continuous variables, parametric data were compared between groups using the *t*‐test, and non‐parametric data were assessed using the Mann–Whitney U test for independent samples. The chi‐square test was employed for the analysis of categorical data to evaluate group differences. To address missing data within our study, we used the mean imputation method, where missing values were replaced by the mean of the observed data within the same variable.

The PSM, initially introduced by Rosenbaum and Rubin^32^ in 1983, is a statistical technique designed to address confounding factors in observational studies.^33^ In this study, we utilized Stata SE version 16.0 to perform PSM, estimating the propensity score with a logistic regression model that incorporated a range of patient characteristics, including age, sex, laboratory data within 24 h of hospital admission, APACHE‐II scores, GCS scores, PCAC and primary disease. We employed proximal‐neighbor PSM without replacement to match patients who underwent ET with those who underwent LT and set the calliper width to 0.2. The quality of matching was evaluated by calculating the standardized differences (StDiff) for selected variables, with an StDiff <0.10 indicating a high degree of matching.^34^ After PSM, we conducted analyses to compare categorical variables between the ET and LT groups using the chi‐square test. For continuous variables, we employed the Mann–Whitney *U* test for non‐parametric data and the t‐test for parametric data, as appropriate. The Kaplan–Meier method was applied to estimate event rates over time to assess survival outcomes. Furthermore, we conducted a sensitivity analysis of the receiver operating characteristic (ROC) curve to rigorously assess the discriminatory power of key predictors identified in our study. Statistical significance was set at a *p*‐value <.05.

## 4 RESULTS

### 4.1 Demographics

**FIGURE 1:**
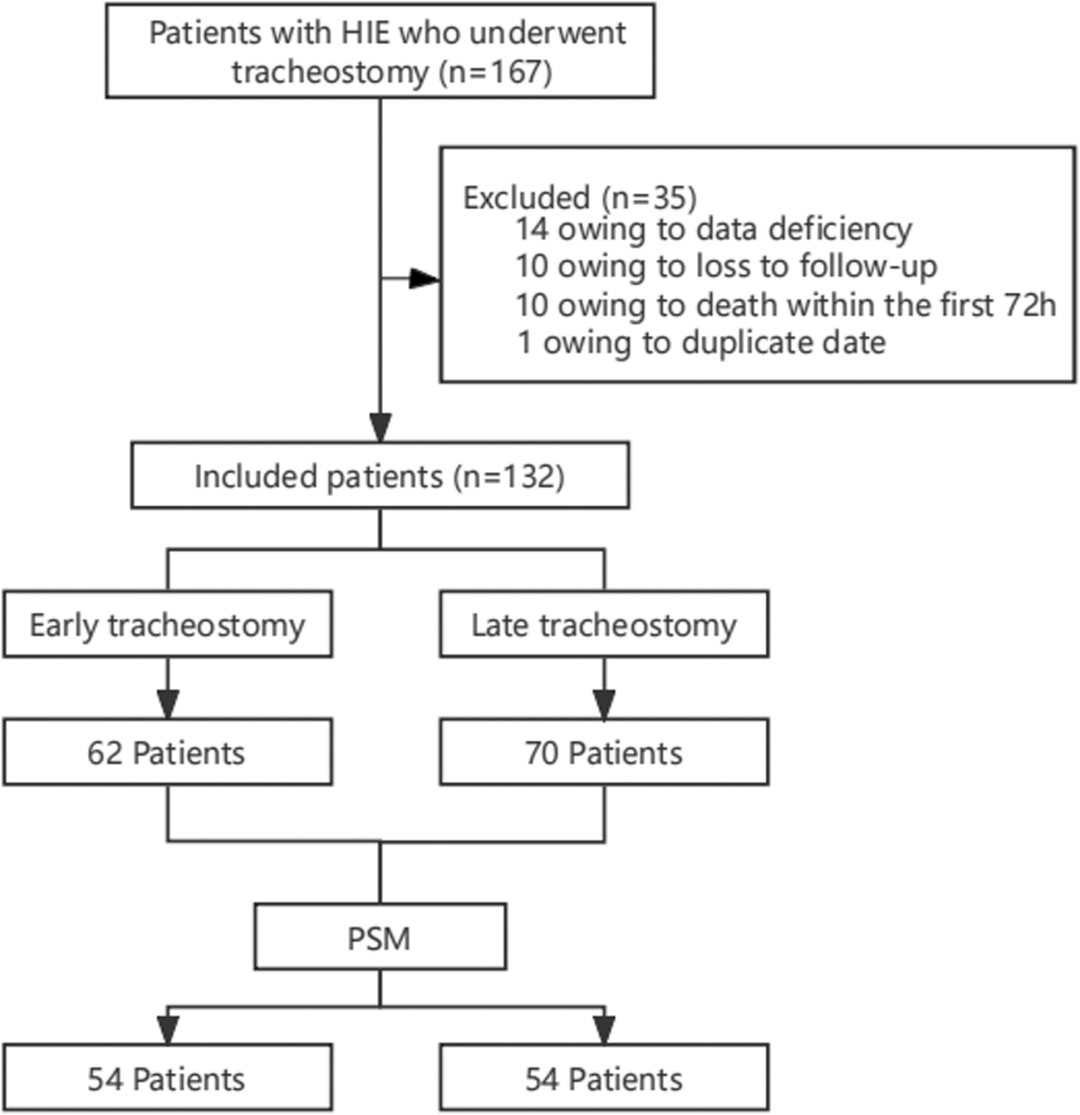
Study design and patient selection. HIE, hypoxic‐ischaemic encephalopathy; PSM, propensity score matching.

Between January 2012 and September 2020, 167 patients with HIE underwent a tracheostomy, 132 of whom met the inclusion criteria. There were 62 patients in the ET group and 70 in the LT group, with an ET rate of 46.97% (Figure 1).

The patient characteristics are summarized in Table 1. There were no significant differences in age or sex between the two groups. The most common primary diseases in the ET versus LT groups were pulmonary disease (7 [11.3%] vs. 17 [24.3%]) and brain injuries (13 [21%] vs. 6 [8.6%]). In disease‐specific severity, the APACHE‐II scores of patients with HIE were 21.48 (6.25) in the ET group and 23.21 (6.32) in the LT group, and the differences were statistically significant (*p* = .017). There were no significant differences in the GCS scores or PCAC. The median GCS scores were 5.5 (IQR, 4–10) in the ET group and 6.5 (IQR, 5–9.25) in the LT group. The PCAC in the ET versus LT group were as follows: I, 8 (12.9%) versus 5 (7.1%); II, 7 (11.3%) versus 17 (24.3%); III, 18 (29%) versus 15 (21.4%); and IV, 29 (46.8%) versus 33 (47.1%).

**TABLE 1:** Baseline characteristics of the patients in the early tracheostomy and late tracheostomy groups before and after propensity score matching.

Variable		Total cohort					Matched cohort				
		ET group (*n* = 62)	LT group (*n* = 70)	Statistics	StDiff	*p* value	ET group (*n* = 54)	LT group (*n* = 54)	Statistics	StDiff	*p* value
Sex (*n*, %)	Male	43 (69.4)	43 (61.4)	χ^2^ = 0.910	0.17	.340	36 (66.7)	32 (59.3)	χ^2^ = 0.635	0.21	.425
	Female	19 (30.6)	27 (38.6)				18 (33.3)	22 (40.7)			
Age (mean [SD], years)		53.71 (19.29)	58.49 (17.70)	T = −1.483	0.26	.479	55.11 (19.53)	58.93 (18.15)	T = −1.052	0.21	.295
Weight (median [IQR], kg)		59 (50–68)	61.5 (50–71.13)	Z = −0.815		.415	59 (50.75–68.5)	63.5 (50–70)	Z = −0.576		.565
Primary disease (*n*, %)	Pulmonary disease	7 (11.3)	17 (24.3)	χ^2^ = 16.782	0.35	.115	6 (11.1)	12 (22.2)	χ^2^ = 11.326	0.30	.416
	Coronary artery disease	6 (9.7)	4 (5.7)				6 (11.1)	4 (7.4)			
	Heart failure	2 (3.2)	8 (11.4)				2 (3.7)	6 (11.1)			
	Stroke	5 (8)	4 (5.7)				5 (9.3)	3 (5.6)			
	Diseases of the musculoskeletal system	4 (6.5)	4 (5.7)				4 (7.4)	3 (5.6)			
	Brain injury	13 (21)	6 (8.6)				11 (20.4)	5 (9.3)			
	Others	25 (40.3)	27 (38.6)				20 (37)	21 (38.9)			
Blood platelet count at hospital admission (median (IQR), mmol/L)		163 (107.25–202.25)	147.5 (92.5–225.75)	Z = −0.283	0.18	.777	164 (108.75–202.25)	160.5 (99–230.25)	Z = −0.123	0.10	.902
Haemoglobin content at hospital admission (mean (SD), mmol/L)		126.23 (30.52)	112.21 (26.01)	T = 2.847	0.49	.005	122.17 (29.14)	118.02 (19.73)	T = 0.866	0.27	.388
Creatinine content at hospital admission (median (IQR), mmol/L)		76.25 (63–101)	79.5 (66.83–141.5)	Z = −1.227	0.16	.220	79.75 (63–104.25)	75 (64–96)	Z = −0.335	0.04	.738
Albumin content at hospital admission (mean (SD), mmol/L)		37.33 (7.95)	36.15 (8.01)	T = 0.845	0.15	.290	36.70 (7.92)	35.75 (6.40)	T = 0.684	0.08	.495
Urea nitrogen content at hospital admission (median (IQR), mmol/L)		6.78 (4.27–10 .11)	6.85 (4.88–11.8)	Z = −0.871	0.02	.384	6.53 (4.15–10.10)	6.48 (4.81–8.6)	Z = −0.258	0.07	.796
GCS score at hospital admission (median (IQR))		5.5 (4–10)	6.5 (5–9.25)	Z = −0.777	0.20	.437	6 (4.75–10)	6.5 (5–10.5)	Z = −0.500	0.10	.617
APACHE‐II score at hospital admission (mean (SD))		21.48 (6.25)	23.21 (6.32)	T = −1.579	0.43	.017	21.98 (6.30)	22.31 (6.40)	T = −0.273	0.10	.786
PCAC at hospital admission(n, %)	*I*	8 (12.9)	5 (7.1)	χ^2^ = 4.923	0.22	.178	8 (14.8)	3 (5.6)	χ^2^ = 7.107	0.29	.069
	*II*	7 (11.3)	17 (24.3)				5 (9.3)	14 (25.9)			
	*III*	18 (29)	15 (21.4)				16 (29.6)	12 (22.2)			
	*IV*	29 (46.8)	33 (47.1)				25 (46.3)	25 (46.3)			
LOS before ICU admission (median (IQR), days)		5.5 (2.75–9.25)	5.5 (1–14.25)	Z = −0.274		.784	5.5 (3–8.25)	5 (1–11.25)	Z = −0.290		.772
The time between the onset and resumption of spontaneous circulation (median (IQR), min)		8.5 (0–18.5)	1 (0–20)	Z = −0.362		.717	8.5 (0–19.5)	4 (0–20)	Z = −0.491		.624
Tracheostomy before ICU admission (*n*, %)	Yes	11 (17.7)	5 (7.1)	χ^2^ = 3.468		.063	10 (18.5)	1 (1.9)	χ^2^ = 8.199		.065
	No	51 (82.3)	65 (92.9)				44 (81.5)	53 (98.1)			
Types of ICU (*n*, %)	Coronary care unit	0 (0)	4 (5.7)	χ^2^ = 19.832		.099	0 (0)	4 (7.4)	χ^2^ = 16.896		.204
	Cardiac ICU	30 (48.4)	42 (60)				25 (46.3)	30 (55.6)			
	Neurological ICU	13 (21)	7 (10)				11 (20.4)	7 (13)			
	Respiratory ICU	2 (3.2)	7 (10)				2 (3.7)	5 (9.3)			
	Surgical ICU	8 (12.9)	4 (5.7)				7 (13)	3 (5.6)			
	Others	9 (14.5)	6 (8.6)				9 (16.7)	5 (9.3)			
Causes of heart attack (*n*, %)	Hypoxemia	24 (38.7)	25 (35.7)	χ^2^ = 3.733		.589	22 (40.7)	19 (35.2)	χ^2^ = 5.705		.336
	Bleeding	13 (21)	16 (22.9)				11 (20.4)	14 (25.9)			
	Ventricular fibrillation	2 (3.2)	7 (10)				2 (3.7)	6 (11.1)			
	Poisoning	1 (1.6)	1 (1.4)				0 (0)	1 (1.9)			
	Others	22 (35.5)	21 (30)				19 (35.2)	14 (25.9)			
The site of the heart attack (*n*, %)	In hospital	24 (38.7)	28 (40)	χ^2^ = 0.023		.880	22 (40.7)	24 (44.4)	χ^2^ = 0.151		.697
	Out of hospital	38 (61.3)	42 (60)				32 (59.3)	30 (55.6)			
Intubation during cardiopulmonary resuscitation (*n*, %)	Yes	40 (64.5)	43 (61.4)	χ^2^ = 0.134		.714	35 (64.8)	35 (64.8)	χ^2^ = 0.000		1.000
	No	22 (35.5)	27 (38.6)				19 (35.2)	19 (35.2)			
Thrombolytic therapy before hospital admission (*n*, %)	Yes	0 (0)	2 (2.9)	χ^2^ = 0.1799		.180	0 (0)	2 (3.7)	χ^2^ = 2.038		.153
	No	62 (100)	68 (97.1)				54 (100)	52 (96.3)			
Hypothermia treatment before hospital admission (*n*, %)	Yes	5 (8.1)	2 (2.9)	χ^2^ = 1.775		.183	5 (9.3)	2 (3.7)	χ^2^ = 1.375		.241
	No	57 (91.9)	68 (97.1)				49 (90.7)	52 (96.3)			
Coronary angiography before ICU admission (*n*, %)	Yes	1 (1.6)	4 (5.7)	χ^2^ = 1.518		.218	1 (1.9)	4 (7.4)	χ^2^ = 1.887		.169
	No	61 (98.4)	66 (94.3)				53 (98.1)	50 (92.6)			
Hypothermia treatment before ICU admission (*n*, %)	Yes	9 (14.5)	9 (12.9)	χ^2^ = 0.077		.782	9 (16.7)	8 (14.8)	χ^2^ = 0.070		.792
	No	53 (85.5)	61 (87.1)				45 (83.3)	46 (85.2)			
Tracheostomy approach (*n*, %)	Open surgery	33 (53.2)	31 (44.3)	χ^2^ = 1.052		.305	30 (55.6)	25 (46.3)	χ^2^ = 0.926		.336
	Percutaneous dilatation tracheostomy	29 (46.8)	39 (55.7)				24 (44.4)	29 (53.7)			
Surgical site (*n*, %)	The operating room	14 (22.6)	19 (27.1)	χ^2^ = 0.365		.546	13 (24.1)	14 (25.9)	χ^2^ = 0.049		.824
	Bedside operation	48 (77.4)	51 (72.9)				41 (75.9)	40 (74.1)			

Abbreviations: APACHE‐II, Acute Physiology and Chronic Health Evaluation‐II; ET, early tracheostomy; GCS, Glasgow Coma Scale; ICU, intensive care unit; IQR, interquartile range; LOS, length of stay; LT, late tracheostomy; PCAC, Pittsburgh Cardiac Arrest Category; SD, standard deviation; StDiff, standardized differences.

Before PSM, the differences in haemoglobin content (*p* = .005; StDiff = 0.49) and APACHE‐II scores (*p* = .017; StDiff = 0.43) between the two groups were statistically significant. Following PSM, we selected 54 pairs of patients with balanced baseline characteristics. In the matched cohort, the haemoglobin content in patients with HIE was 122.17 (29.14) in the ET group and 118.02 (19.73) in the LT group (*p* = .388; StDiff = 0.27). The APACHE‐II scores in patients with HIE were 21.98 (6.30) in the ET group and 22.31 (6.40) in the LT group (*p* = .786; StDiff = 0.10) (Table 1). The baseline data were balanced between the two groups.

### 4.2 Primary clinical outcomes

Following a comparison of the primary clinical outcomes between the ET and LT groups after PSM (Table 2), we observed that the hospital LOS in the ET group (median, 19.5 days; IQR, 10.87–36.5) was significantly shorter than that in the LT group (median, 39.5 days; IQR, 22–66) (*p* < .001). Regarding the ICU LOS, that of the ET group (median, 14.5 days; IQR, 6.75–26) was significantly shorter than that of the LT group (median, 35 days; IQR, 20–59) (*p* < .001). Moreover, the duration of MV was significantly shorter in the ET group (median, 12 days; IQR, 7–20) than in the LT group (median, 28 days; IQR, 15.75–58.25) (*p* < .001).

**TABLE 2:** Comparison of the outcomes of the early tracheostomy and late tracheostomy groups before and after propensity score matching.

Variable		Total cohort				Matched cohort			
		ET group (*n* = 62)	LT group (*n* = 70)	Statistics	*p* value	ET group (*n* = 54)	LT group (*n* = 54)	Statistics	*p* value
Successful weaning from the ventilator following a tracheostomy (*n*, %)	Yes	26 (41.9)	34 (48.6)	*χ* ^2^ = 0.584	.445	22 (40.7)	29 (53.7)	χ^2^ = 1.820	.177
	No	36 (58.1)	36 (51.4)			32 (59.3)	25 (46.3)		
Incidence of VAP (*n*, %)	Yes	5 (8)	4 (5.7)	*χ* ^2^ = 1.338	.247	3 (5.6)	4 (7.4)	*χ* ^2^ = 0.901	.343
	No	57 (92)	66 (94.3)			51 (94.4)	50 (92.6)		
Duration of MV (median [IQR], days)		12 (7–20)	30.5 (17–62.25)	Z = −5.272	< .001	12 (7–20)	28 (15.75–58.25)	Z = −4.704	<.001
ICU LOS (median [IQR], days)		14 (6.75–26)	40.5 (21.5–64.25)	Z = −5.781	< .001	14.5 (6.75–26)	35 (20–59)	Z = −4.927	<.001
Hospital LOS (median [IQR], days)		19 (9.75–36.5)	47 (24–70.25)	Z = −5.757	< .001	19.5 (10.87–36.5)	39.5 (22–66)	Z = −4.241	<.001
Discharge status (*n*, %)	Death	16 (25.8)	14 (20)	*χ* ^2^ = 0.648	.723	14 (25.9)	9 (16.7)	*χ* ^2^ = 1.382	.501
	Voluntary discharge	29 (46.8)	36 (51.4)			25 (46.3)	28 (51.9)		
	Recovery	17 (27.4)	20 (28.6)			15 (27.8)	17 (31.5)		
Inhospital mortality (*n*, %)	Survival	29 (46.8)	34 (48.6)	*χ* ^2^ = 0.043	.837	23 (42.6)	29 (53.7)	*χ* ^2^ = 1.335	.248
	Death	33 (53.2)	36 (51.4)			31 (57.4)	25 (46.3)		
30‐day all‐cause mortality (*n*, %)	Survival	28 (45.2)	34 (48.6)	*χ* ^2^ = 0.154	.695	22 (40.7)	29 (53.7)	*χ* ^2^ = 1.820	.177
	Death	34 (54.8)	36 (51.4)			32 (59.3)	25 (46.3)		
1‐year all‐cause mortality (*n*, %)	Survival	26 (41.9)	33 (47.1)	*χ* ^2^ = 0.361	.548	21 (38.9)	28 (51.9)	χ^2^ = 1.831	.176
	Death	36 (58.1)	37 (52.9)			33 (61.1)	26 (48.1)		

Abbreviations: ET, early tracheostomy; ICU, intensive care unit; IQR, interquartile range; LOS, length of stay; LT, late tracheostomy; VAP, ventilator‐associated pneumonia; MV, mechanical ventilation.

### 4.3 Secondary outcomes

After PSM, seven patients developed VAP, with a lower incidence in the ET group than in the LT group (5.6% vs. 7.4%); however, the difference was not statistically significant (*p* = .343). The 30‐day mortality rates (59.3% vs. 46.3%, *p* = .177), inhospital mortality rates (57.4% vs. 46.3%, *p* = .248) and 1‐year mortality rates (61.1% vs. 48.1%, *p* = .176) in the ET group were higher than those in the LT group; however, the differences were not statistically significant.

**FIGURE 2:**
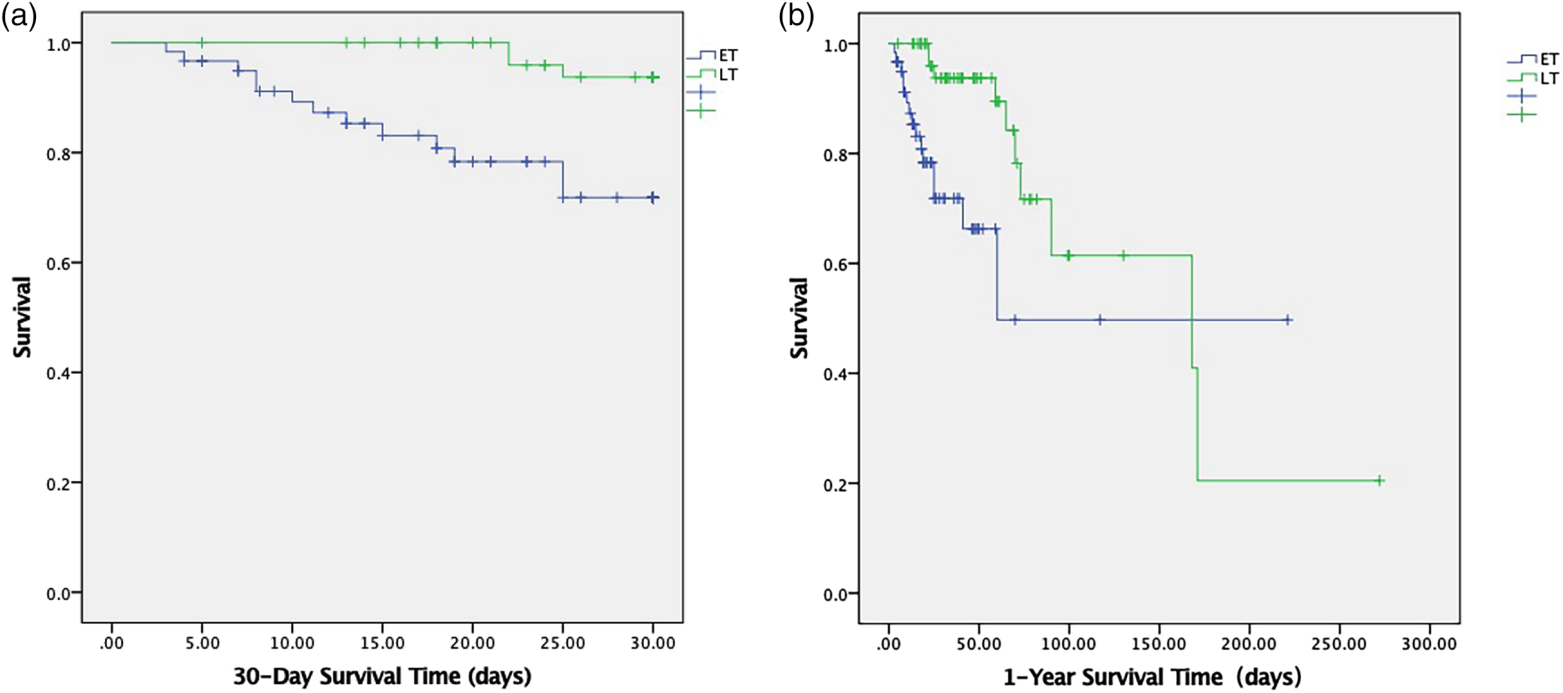
(a) 30‐day survival time. (b). 1‐year survival time. (a, b) Kaplan–Meier survival curves. The *y*‐axis represents the cumulative survival function, while the *x*‐axis represents the survival time (days). ET, early tracheostomy; LT, late tracheostomy.

Subsequently, a survival analysis was conducted. Figure 2a shows a statistically significant difference in the 30‐day survival time between the ET and LT groups, with the risk of death in the ET group being 6.175 times higher than that in the LT group (95% CI, 1.752–21.765; *p* = .005). Figure 2b shows a statistically significant difference in the 1‐year survival time between the ET and LT groups, with the risk of death in the ET group being 3.088 times higher than that in the LT group (95% CI, 1.346–7.085; *p* = .008).

### 4.4 Sensitivity analysis

**FIGURE 3:**
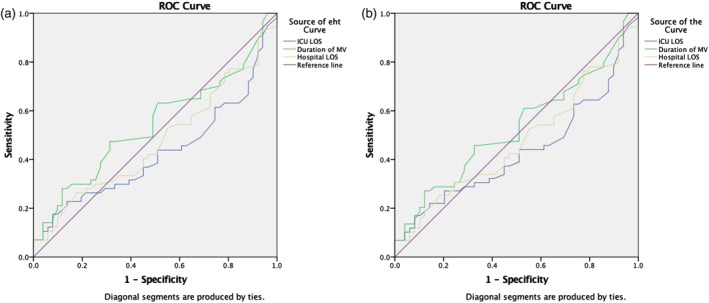
(a) Receiver operating characteristic analysis of the 30‐day mortality. (b) Receiver operating characteristic analysis of the 1‐year mortality. (a, b) Receiver operating characteristic analysis. The y‐axis represents the sensitivity, while the x‐axis represents the specificity. ROC, receiver operating characteristic; ICU, intensive care unit; LOS, length of stay; MV, mechanical ventilation.

We conducted a sensitivity analysis utilizing the ROC curve to assess the predictive capacity of various factors (Figure 3a,b). The 30‐day mortality ROC analysis indicated that the duration of MV was the most significant predictor of mortality, with an area under the curve (AUC) of 0.543 (*p* = .279). Similarly, for 1‐year mortality, the ROC analysis identified the duration of MV as the primary predictor, yielding an AUC of 0.531 (*p* = .440). However, the analysis revealed that ICU LOS (AUC = 0.427, *p* = .195), and hospital LOS (AUC = 0.473, *p* = .650) were not significant predictors of 30‐day mortality in our study cohort. Similarly, ICU LOS (AUC = 0.433, *p* = .173), and hospital LOS (AUC = 0.473, *p* = .510) were not significant predictors of 1‐year mortality. Subsequently, a multivariate analysis was performed on the data. Table 3 demonstrates that the duration of MV significantly impacts 30‐day mortality in both the ET group (odds ratio [OR] = 0.710, 95% confidence interval [CI] = 0.506–0.980, *p* = .038) and the LT group (OR = 0.932, 95% CI = 0.871–0.998, *p* = .043). Similarly, the duration of MV is a critical factor influencing 1‐year mortality in both the ET group (OR = 0.899, 95% CI = 0.818–0.989, *p* = .030) and the LT group (OR = 0.935, 95% CI = 0.876–0.999, *p* = .048), as shown in Table 4. In contrast, neither ICU LOS, nor hospital LOS was associated with an increased risk of 30‐day or 1‐year mortality (Tables 3, 4).

**TABLE 3:** Multivariate analysis of the 30‐day mortality.

	*p*‐value	Odds ratio	95% confidence interval for the odds ratio	
			Lower	Upper
ET				
Intercept	.405	0.607	0.187	1.968
Duration of MV	.038	0.710	0.506	0.980
ICU LOS	.067	1.347	1.010	1.795
Hospital LOS	.760	1.013	0.935	1.098
LT				
Intercept	.285	3.381	1.136	10.063
Duration of MV	.043	0.932	0.871	0.998
ICU LOS	.196	1.048	0.976	1.126
Hospital LOS	.636	0.989	0.943	1.036

Abbreviations: ET, early tracheostomy; ICU, intensive care unit; LOS, length of stay; LT, late tracheostomy; MV, mechanical ventilation.

**TABLE 4:** Multivariate analysis of the 1‐year mortality.

	*p*‐value	Odds ratio	95% confidence interval for the odds ratio	
			Lower	Upper
ET				
Intercept	.078	0.466	0.199	1.090
Duration of MV	.030	0.899	0.818	0.989
ICU LOS	.132	1.086	0.975	1.208
Hospital LOS	.650	1.018	0.943	1.097
LT				
Intercept	.064	2.725	0.944	7.869
Duration of MV	.048	0.935	0.876	0.999
ICU LOS	.220	1.050	0.974	1.121
Hospital LOS	.730	0.992	0.947	1.039

Abbreviations: ET, early tracheostomy; ICU, intensive care unit; LOS, length of stay; LT, late tracheostomy; MV, mechanical ventilation.

## 5 DISCUSSION

This study investigated the outcomes following ET and LT in patients with HIE requiring an artificial airway. Our findings demonstrated that among patients with HIE, ET correlated with a reduction in the duration of MV, ICU LOS and the overall hospital LOS. Although the ET group exhibited higher short‐ and long‐term mortality rates than did the LT group, these differences did not reach statistical significance. Our results align with the guidelines of the Federation of Societies of Critical and Intensive Therapy Medicine,^35^ which suggest that ET may contribute to a decrease in the duration of MV without a concomitant reduction in long‐term mortality rates.

PSM is a method employed to mitigate selection bias in observational studies, thereby enhancing the validity of comparative analyses. In our study, we utilized this method to adjust for disease severity and to compare the outcomes of ET and LT, even with the limitations posed by the modest amount of retrospective data available.

In this observational study based on PSM, we found that ET was associated with a reduced duration of MV. Two retrospective studies also suggested that the benefits of ET on the duration of MV represent a reasonable strategy.^36^, ^37^ Furthermore, a PSM cohort study^38^ has reported that ET is associated with a decreased duration of MV in patients with traumatic brain injury. However, in the PSM analysis conducted in that study, only the GCS score was utilized as an indicator of illness severity, which may not fully capture the complexity of confounding factors that could influence the outcomes. Additionally, two studies^20^, ^39^ have reported similar conclusions regarding the benefits of ET in reducing the duration of MV. However, these analyses also acknowledged sex and age disparities among the study populations, as well as significant heterogeneity across the included studies. Such factors could introduce confounding biases that may affect the interpretation of the results. In contrast, a meta‐analysis^40^ demonstrated that ET did not significantly improve the incidence of pneumonia, duration of MV or ICU LOS. The divergent conclusions drawn from various studies may stem from differences in demographic factors, such as sex and age, as well as the inherent heterogeneity across study designs and patient populations. To mitigate biases related to disease severity and enhance the reliability of our findings, the present study employed PSM. This method helps control for confounding variables, thereby providing a more balanced comparison between groups and providing evidence for the creation of long‐term rehabilitative care programmes for ICU survivors.

In this study, there was no significant difference in the incidence of VAP between the two groups. For patients requiring prolonged MV, ET is beneficial for controlling lung infections, reducing the duration of MV and decreasing the incidence of VAP.^18^, ^41^, ^42^, ^43^ A meta‐analysis^17^ found that ET improved VAP and respiratory outcomes but did not improve short‐term overall mortality. Furthermore, Diaz‐Prieto et al.^26^ proposed that ET not only shortened the duration of MV and ICU LOS but also reduced the sedation time. VAP is a major contributor to the increased consumption of health care resources and elevated treatment costs.^44^ ET has demonstrated the potential to alleviate the economic burden for patients by reducing the incidence of VAP, thereby impacting the cost‐effectiveness of care.^45^ However, it is important to note that the current body of research predominantly rates critically ill patients, with a dearth of subspecialized research evidence, particularly concerning patients with HIE. This underscores the need for further investigation into the nuances of HIE and the development of targeted treatment strategies that can improve patient outcomes in this specific population.

In our study, we found that ET was associated with a reduction in both ICU and hospital LOS. A previous study by Hosokawa et al.^46^ showed that patients undergoing ET had a 65% reduction in total ICU LOS compared to that in critically ill patients undergoing LT treatment. Studies^47^, ^48^ conducted on neurosurgical and critically ill patients with stroke also confirmed that ET significantly reduced the ICU LOS. Although the differences were not statistically significant, a previous study^36^ revealed that regarding patients in the ICU, the hospital LOS in the ET group was shorter than that in the LT group. However, this difference was not statistically significant. Furthermore, several systematic reviews^17^, ^18^, ^20^, ^22^ based on RCTs have found that patients undergoing ET have significantly shorter ICU LOS than do those undergoing LT. In a propensity‐matched cohort study^37^ involving patients with traumatic brain injury, ET correlated with a reduced duration of MV, ICU LOS and hospital LOS, yet it did not significantly influence mortality rates. Patients with more severe injuries might require more extensive treatment, potentially leading to a delay in tracheostomy and longer expected hospital stays because of poorer outcomes. The observed trend in the ET group may suggest potential benefits in terms of recovery and expedited discharge. However, these observations require further investigation through rigorous research to confirm their validity and generalizability.

Despite observing a higher mortality rate in the ET group than in the LT group, our study did not identify statistically significant differences in the 30‐day, 1‐year or inhospital mortality rates between the two groups. However, the survival analysis conducted as part of this research suggested a superior survival outcome for the LT group at both the 30‐day and 1‐year follow‐up intervals when compared to the ET group. Two meta‐analyses^17^, ^49^ also indicated that ET in ICU patients is not significantly associated with an altered risk of mortality. A recent meta‐analysis^50^ of adult patients without neurological injuries reported no significant difference in overall mortality rates between the ET and LT groups. Consistent conclusions were reached in two additional meta‐analyses^18^, ^51^ involving critically ill adult patients. Moreover, a Cochrane review^7^ that included critically ill patients and drew evidence from 12 RCTs found no significant reduction in either short‐term or long‐term mortality rates in the ET group compared to the LT group. However, a network meta‐analysis^16^ suggested that performing tracheostomy within 4 days after intubation was associated with a lower short‐term mortality rate than was performing tracheostomy at or after 13 days post‐intubation. The potential advantages of ET are further supported by evidence from RCTs,^52^ indicating that patients undergoing LT may be at a higher risk of adverse neurological outcomes, which could contribute to increased mortality. In our study, we meticulously matched covariates using PSM, including age, sex, GCS scores, APACHE‐II scores, PCAC and various laboratory parameters. This methodological approach was employed to mitigate the influence of potential confounders and enhance the comparability of the groups under investigation. Nonetheless, it is important to acknowledge that the factors impacting the mortality of critically ill patients are both multifaceted and intricate. Thus, the association between ET and mortality warrants further in‐depth research to fully understand the nuances of this relationship.

### 5.1 Implications for practice

ET has been shown to reduce the duration of MV, ICU LOS and overall hospital LOS. However, its impact on mortality remains a subject of debate. Extending the follow‐up period in future studies could provide a clearer understanding of how different timings of tracheostomy affect the long‐term prognosis of patients. Moreover, future research must consider factors that are pertinent to clinicians and local health care systems. To address this, rigorously designed, large‐sample RCTs may be required to ascertain the potential survival benefits of ET for patients with HIE. Such trials will be instrumental in enabling clinicians to evaluate functional prognoses, enhance clinical care and facilitate the optimization of medical resource allocation.

### 5.2 Limitations

This study had some limitations. First, the present study's single‐centre retrospective design, with limited patient participation, may have introduced selection bias. Consequently, the generalizability of our findings may be limited. Second, our focus on key variables for propensity scoring may have inadvertently excluded other influential factors. For instance, the use of sedative drugs in patients with brainstem lesions could significantly affect patient outcomes because of potential respiratory depressive side effects. Additionally, the study did not include patient comfort as a variable, an oversight that highlights the need for a more holistic approach in future research to include patient‐reported outcomes. Third, the lack of standardized definitions of ‘early’ and ‘late’ tracheostomy across studies complicates result comparisons. Although some studies^53^, ^54^ suggest that tracheostomy should be performed approximately 10 days after endotracheal intubation, the optimal timing of this procedure remains a subject of debate. A standardized approach is needed in future research to ensure more reliable and valid comparisons. Fourth, the relatively short follow‐up period in our study limits the understanding of the long‐term effects of ET on patient prognosis and health care resource utilization. Therefore, further research with extended follow‐up periods is essential to assess the long‐term efficacy and implications of tracheostomy in critically ill patients.

## 6 CONCLUSION

Although the timing of tracheostomy does not appear to be significantly correlated with a reduction in VAP occurrence or improvements in mortality rates among patients with HIE, our study indicates that it can lead to a shorter duration of MV, ICU LOS and overall hospitalization LOS. In clinical practice, for patients with HIE at an elevated risk for prolonged MV, the potential of ET to shorten hospital LOS suggests its consideration as a viable treatment option. Despite the challenges in determining the precise indications for ET, our findings underscore the practical advantages of this procedure. However, given the limited sample size of our study, we are unable to definitively exclude the possibility of differences in mortality rates between the ET and LT groups. Further research with larger cohorts is warranted to provide more conclusive evidence on the impact of tracheostomy timing on mortality and other clinical outcomes.

## AUTHOR CONTRIBUTIONS

Yeling Li conceived and designed the study, was responsible for the methodology, analysed the data, prepared the manuscript. Dingyuan Wan conceived and designed the study and collected the data. Hongmei Liu, Keying Guo, Yilin Liu, Lihong Zhao and Ming Li conducted the investigation and collected the data. Jijie Li conducted methodological guidance and statistical analysis. Yiwen Liu and Wei Dong were responsible for project administration and supervision.

## FUNDING INFORMATION

Key Research and Development project fund of the Department of Science and Technology of Sichuan Province, Grant/Award Number: 2023YFQ0099.

## CONFLICT OF INTEREST STATEMENT

The authors declare that there are no conflicts of interest.

## ETHICS STATEMENT

This project was approved by the Ethics Committee of West China Hospital of Sichuan University (December 29, 2021; No.2021‐1651).

## Data Availability

The data that support the findings of this study are openly available in PubMed at https://pubmed.ncbi.nlm.nih.gov/?Db=pubmed.
